# Alteration of the Conformational Dynamics of a DNA Hairpin by α‐Synuclein in the Presence of Aqueous Two‐Phase Systems

**DOI:** 10.1002/chem.202002119

**Published:** 2020-08-06

**Authors:** Sanjib K. Mukherjee, Jim‐Marcel Knop, Simone Möbitz, Roland H. A. Winter

**Affiliations:** ^1^ Physical Chemistry I–Biophysical Chemistry Faculty of Chemistry and Chemical Biology TU Dortmund University Otto-Hahn Str. 4a 44227 Dortmund Germany

**Keywords:** biophysics, DNA hairpin, high pressure chemistry, smFRET, α-synuclein

## Abstract

The effect of an amyloidogenic intrinsically disordered protein, α‐synuclein, which is associated with Parkinson's disease (PD), on the conformational dynamics of a DNA hairpin (DNA‐HP) was studied by employing the single‐molecule Förster resonance energy transfer method. The open‐to‐closed conformational equilibrium of the DNA‐HP is drastically affected by binding of monomeric α‐synuclein to the loop region of the DNA‐HP. Formation of a protein‐bound intermediate conformation is fostered in the presence of an aqueous two‐phase system mimicking intracellular liquid‐liquid phase separation. Using pressure modulation, additional mechanistic information about the binding complex could be retrieved. Hence, in addition to toxic amyloid formation, α‐synuclein may alter expression profiles of disease‐modifying genes in PD. Furthermore, these findings might also have significant bearings on the understanding of the physiology of organisms thriving at high pressures in the deep sea.

## Graphical abstract

**The effect of an amyloidogenic** intrinsically disordered protein, α‐synuclein, on the conformational dynamics of a DNA hairpin was studied by employing the single‐molecule Förster resonance energy transfer method. Using pressure modulation, additional mechanistic information about the binding complex could be retrieved.

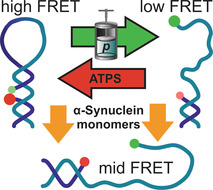

DNA‐protein interactions and the conformational stability of DNA are crucial for normal cell function.^1^ It has been reported that neuro‐proteins, such as α‐synuclein (α‐Syn), which is directly linked with Parkinson's disease, and other amyloidogenic proteins may be involved in the conformational stability of DNA.^2^, ^3^, ^4^, ^5^ However, to what extent and how the local conformational dynamics of DNA is altered upon binding with such neuro‐proteins is still largely unknown.^5^, ^6^ Altered gene expression in most of the human diseases like Parkinson's disease (PD) and Alzheimer's disease (AD) is observed due to conformational changes of the DNA.^7^, ^8^ It has been found that the conformation of the DNA in PD‐affected human postmortem brain cells is altered from the normal B‐form to an altered B‐conformation, and in AD, a conformational transition occurs from the B‐form to the Z‐form.^7^, ^8^ The factors responsible for these conformational fluctuations are still not clear. As α‐Syn acts as a transcription modulator,^9^ it has a significant influence on gene expression profiles, which cause also neuronal cell dysfunction.^9^, ^10^, ^11^ DNA has also been reported to stimulate amyloid formation (fibrillization) in vitro.^10^ Remarkably, only a limited number of studies has been carried out on the interaction between monomeric α‐synuclein and nucleic acids. In this respect, the conformational landscape of non‐canonical DNA structures, such as DNA hairpins (DNA‐HPs), is of particular interest because they regulate gene expression along with playing a significant role in DNA recombination and transposition.^12^, ^13^, ^14^ Hence, these structures are very attractive from a biophysical point of view for investigating the conformational dynamics of α‐Syn‐DNA interactions,^15^, ^16^ which is the purpose of this study.

In recent years, it has become increasingly clear that the physicochemical properties of biomolecules may also get significantly altered in the crowded in vivo situation when compared to those in dilute solution.^17^, ^18^, ^19^, ^20^, ^21^ Furthermore, regulation of biochemical processes is frequently achieved through the compartmentalization of the cellular milieu. In this respect, non‐membrane bound compartments consisting of phase‐separated liquid‐like droplets of proteins and protein‐RNA mixtures have been shown to be of particular importance, which are supposed to significantly alter cellular reactions as well.^22^, ^23^, ^24^ Due to the lack of a physical barrier, such as a lipid membrane, these liquid condensates are able to exchange their components rapidly with the surrounding medium. The effect of liquid–liquid phase separation (LLPS) as observed in artificial aqueous two‐phase systems (ATPS) on the conformational dynamics of biomolecules, including DNA, is hardly explored, however,^25^ and has therefore been included in this study.

To yield a molecular level understanding of the conformational dynamics of α‐Syn interacting with DNA hairpins (DNA‐HPs), single‐molecule Förster resonance energy transfer (smFRET) experiments have been carried out. SmFRET has emerged as one of the most powerful techniques for elucidating dynamical properties of biomolecules as it provides mechanistic information on the underlying molecular level interactions, which are otherwise averaged out in ensemble‐based experiments.

As a consequence of intra‐strand hybridization between complementary sequences, oligonucleotides are able to form hairpin structures, where the duplex region generated upon hybridization forms the stem of the hairpin and the nucleotides in between form the hairpin loop. In solution, equilibrium is established between oligonucleotides in hairpin conformation and oligonucleotides that are not self‐hybridized, establishing an open structure. The chemical environment surrounding the DNA‐HP is expected to influence its hybridization abilities extensively. Here, we focus on applications of smFRET to study the conformational dynamics of a DNA‐HP which contains 32 adenine residues in the loop (Figure 1 A) upon interacting with the disordered monomeric protein α‐Syn in neat buffer and under liquid–liquid phase separation conditions.

**Figure 1:**
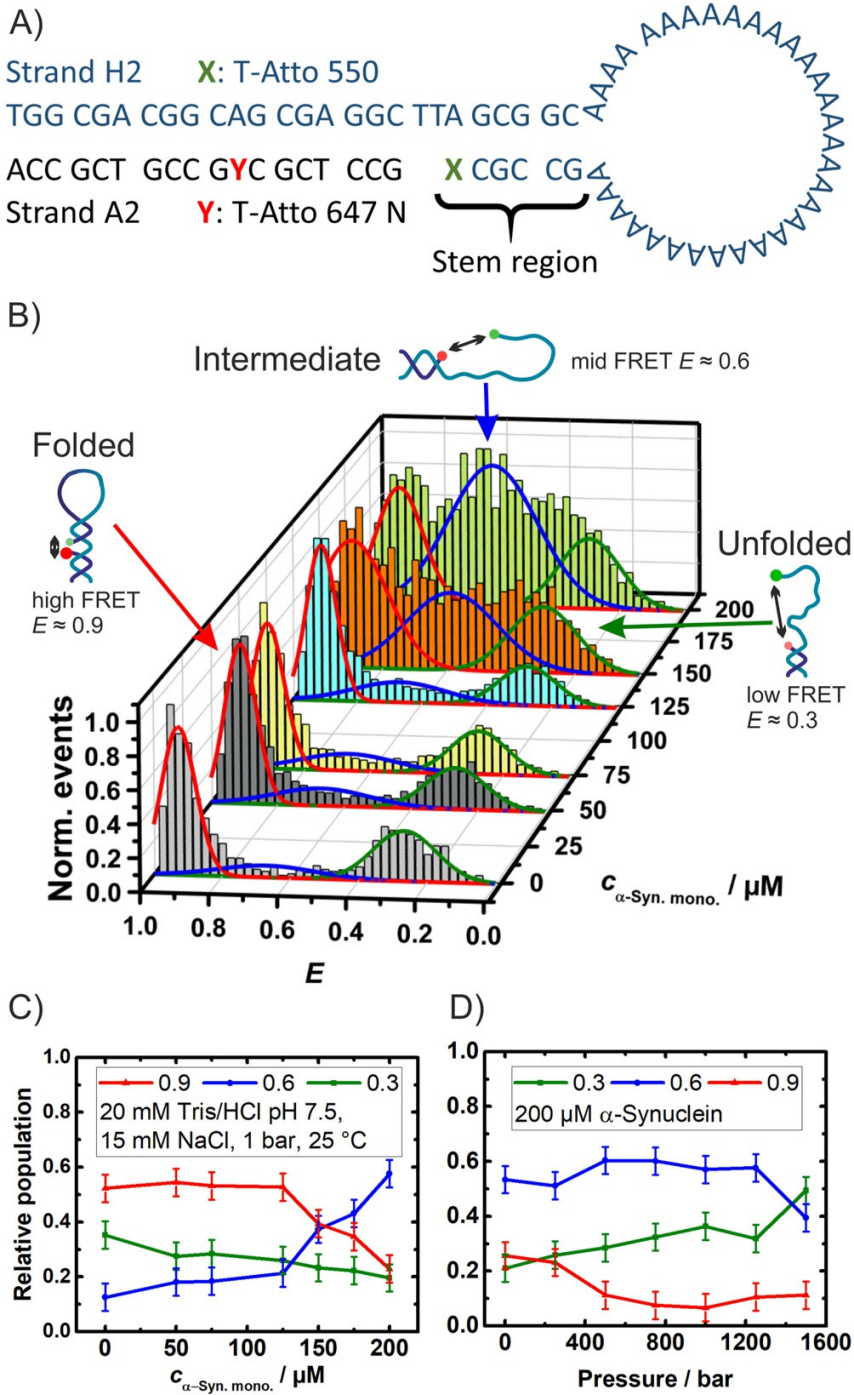
A) Schematic representation of the DNA‐HP′s sequence with the attached fluorophores (FRET pair) Atto 647 N and Atto 550. B) SmFRET‐histograms of the DNA‐HP as a function of the concentration of monomeric α‐Syn. C) Relative population of conformers of the DNA‐HP (calculated using a three Gaussian fit) as a function of monomeric α‐Syn concentration. D) Pressure dependent relative population at 200 μm α‐Syn and the same buffer as in C).

To reveal additional mechanistic details of the interaction process, pressure modulation has been used as well. Next to its biological relevance for understanding the physiology of deep‐sea organisms living at high hydrostatic pressure (HHP) conditions of several hundreds of bar, pressure‐axis experiments have been shown to enable modulation of intra‐ and inter‐molecular interactions and reveal details of the free‐energy and conformational landscape of biomolecules.^26^, ^27^, ^28^, ^29^, ^30^ According to Le Châtelier's principle, an increase of pressure shifts an equilibrium towards the state that occupies the smallest possible overall volume. The pressure effect on a given reversible reaction follows the relation (dln*K*/d*p*)_*T*_=−Δ*V*/(R*T*), where *K* is the pressure‐dependent equilibrium constant and Δ*V* is the associated volume change of the transition, which depends sensitively on the packing and hydration properties of the biomolecule.^26^, ^27^, ^28^, ^29^, ^30^, ^31^

Figure 1 B shows smFRET measurements of the DNA‐HP under freely diffusing conditions in the presence of different concentrations of monomeric α‐Syn (for details of sample preparation and technical aspects, please refer to the Supporting Information). The FRET histograms of the DNA‐HP display two FRET distribution peaks. They are related to conformations with different separations, *R*, of the two attached dyes and thus different FRET efficiencies, *E*, as *E*=R60 /(R60 +*R* ^6^). The Förster radius, that is, the distance at which 50 % of the excited donor molecules will be deactivated, is *R*_0_=6.5nm for the fluorophores used (Atto 550 and Atto 647 n).^25^, ^32^ The two peaks are located at *E*≈0.3 and *E*≈0.9, respectively. The peak at the lower FRET efficiency represents the open state, where the donor and acceptor distance is maximum, and the higher FRET efficiency represents the closed conformation of the DNA‐HP, where donor and acceptor are at a proximal distance. At ambient temperature and pressure conditions, the ratio of the open to closed state is approximately 0.81.^33^

As shown in Figure 1 B, with increasing α‐Syn concentration a further broad peak centered at *E* values around 0.5 to 0.6 emerges in the FRET histograms, which points to a population of intermediate states of the DNA‐HP induced by non‐specific interactions with the monomeric α‐Syn. The fractions of open, intermediate and closed conformations can be determined from the respective area under the curve for each distribution (Figure 1 C).

Recently, it has been shown that HHP application on the DNA‐HP in neat buffer solution gradually populates the low‐FRET distribution species (*E*≈0.3), which is due to the unfolding of the DNA‐HP.^25^, ^32^, ^33^ Figures 1 D and Figure 2 display the pressure effect on the conformational states of the DNA‐HP in the presence of different concentrations of α‐Syn (the smFRET histograms are shown in Figure S1). From these and literature data for the DNA‐HP system in neat buffer solution (Figures 3 and 4 in Ref. 25), it is evident that the open conformation (unfolded state) becomes more populated with increasing pressure in the absence^25^ and presence of α‐Syn (Figure 2).The intermediate conformation of the DNA‐HP remains essentially unaffected by pressure, pointing to formation of a compact, void‐free and pressure‐stable DNA‐HP‐α‐Syn complex. Taking into account the open and closed conformation only, within the accuracy of the data, a volume change in the order of that in neat buffer solution (Δ*V=*−27 cm^3^ mol^−1^)^25^ can be calculated for unfolding of the DNA‐HP.

**Figure 2:**
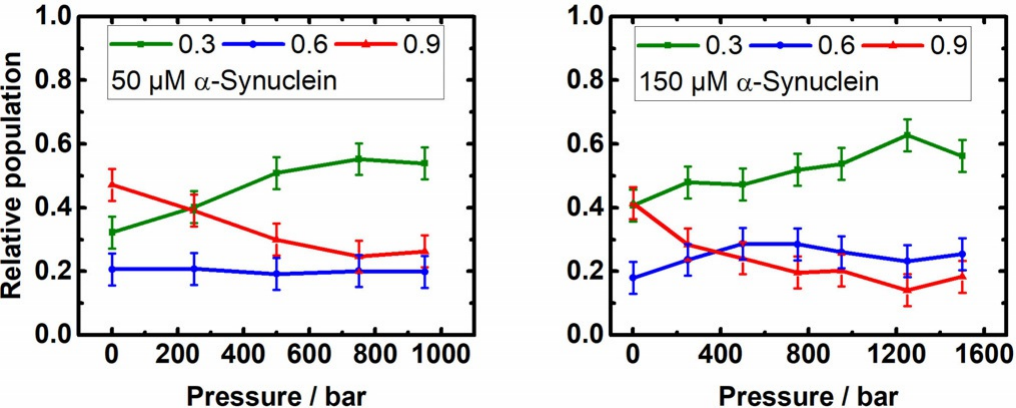
Relative population of the DNA‐HP conformations as a function of pressure. The concentration of monomeric α‐Syn was 50 μm (left) and 150 μm (right). Buffer conditions were 20 mm Tris/HCl pH 7.5, 15 mm NaCl, *T*=25 °C; *E*: 0.3 unfolded/open, 0.6 intermediate, 0.9 folded/closed.

LLPS is known to significantly modulate an array of physiological processes, including protein dynamics, folding, aggregation, and activity, to name a few.^23^, ^25^, ^36^, ^37^ Here, we used an artificial ATPS composed of a dextran (11 wt %) and PEG (11 wt %) mixture to mimic intracellular LLPS conditions. The advantage of this system is the absence of a pressure effect on the stability of the ATPS itself.^25^ In a previous study, we could show that the DNA‐HP partitions inside the dextran‐rich droplets of the ATPS (Figure 1 in Ref. 25) and that the ATPS significantly modulates the pressure‐dependence of the conformational dynamics of the DNA‐HP.^25^ Partitioning of the DNA‐HP in the ATPS markedly counteracts the effect of pressure‐induced destabilization of the closed conformation.^25^

Conversely, here, in the presence of α‐Syn at all concentrations measured (from 50 to 200 μm), the FRET efficiency distribution data of the DNA‐HP at 50 μm α‐Syn concentration exhibits a pronounced maximum at *E*≈0.60, indicating stabilization of the intermediate conformation (Figure 3 and Figure S1), which reaches even ≈90 % of the overall distribution, independent of the concentration of α‐Syn. Further, this population remains essentially unchanged upon pressure application. Upon addition of α‐Syn, partitioning of the DNA‐HP is changing from the dextran‐rich droplet phase to the PEG‐rich phase, which contains about 14 % PEG.^36^

**Figure 3:**
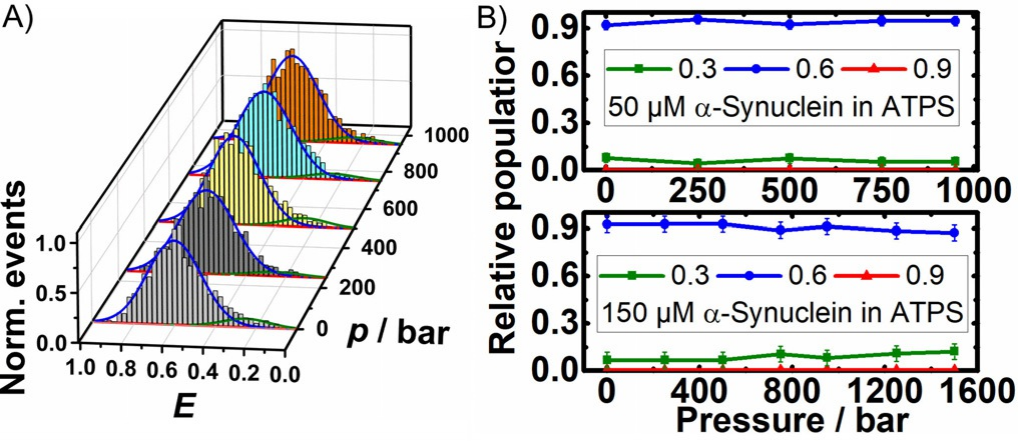
SmFRET histogram of the DNA‐HP (A) and relative population of the DNA‐HP′s conformations as a function of pressure (B). The concentration of monomeric α‐Syn was 50 μm in A) and 50 and 150 μm in B). Solution conditions were 11 wt % PEG + 11 wt % Dextran (ATPS), 20 mm Tris/HCl pH 7.5, 15 mm NaCl, *T*=25 °C; *E*: 0.3 unfolded/open, 0.6 intermediate, 0.9 folded/closed.

As revealed by complementary measurements in 30 wt % dextran and in 11 wt % PEG, serving as simple crowding agents only, at concentrations near to those encountered in the different phases of the ATPS (Figures S2 and S3), the ratio of folded and intermediate conformations is shifted to the folded species when compared with the corresponding ATPS data (Figure 3). The change from approximately 50 % folded and 30 % intermediate states in the neat crowders to about 0 % folded and 90 % intermediate conformers in the PEG‐rich phase of the ATPS is quite dramatic. These data suggest that the molecular properties of the PEG‐rich phase in the ATPS are different from those of the crowding agents alone. Such differences of DNA‐protein interactions in the presence of droplet condensates of the ATPS compared to simple crowding agents may be of importance for understanding biochemical processes in cellulo.

Intrinsically disordered proteins that bind to nucleic acids with high affinity represent a genetically controllable strategy for modulating the conformation and dynamics of nucleic acids in a cellular environment. Collectively, our data reveal that α‐Syn is able to alter the structural pattern and formation of DNA‐HPs, thereby changing the conformational dynamics and stabilizing altered conformations, which may have significant biological effects on the gene expression patterns.

The amino acid sequence of α‐Syn can be partitioned into three regions, namely, 1) the N‐terminal region (residues 1–60) which contains four regions of 11 imperfect repeats with the KTKGEV consensus sequence and is responsible for interaction with negatively charged biomolecules such as lipids and nucleic acid backbones, 2) the central NAC region (residues 61–95), showing high sequence hydrophobicity that has been implicated in the aggregation and amyloid formation of the protein, and 3) the C‐terminal region (residues 96–140), which is very acidic, containing ten glutamate and five aspartate residues and predominantly hydrophilic, its role still not being clearly understood.^37^, ^38^, ^39^

It has been shown that α‐Syn is able to bind to DNA double strands and that upon protein binding the DNA persistence length increases, but base‐pairing does not seem to be disturbed.^10^ However, its interaction with oligonucleotides and non‐canonical DNA structures is still largely unknown. We show that also unaggregated monomeric α‐Syn has a severe effect on the dynamical events of DNA hairpins, largely affecting their conformational transition between the closed and the open, non‐selfhybridized conformation, and inducing alternate conformations upon protein binding. This is in line with results of Naraynan et al.,^40^ showing that substates of nucleic acids are separated by low free energy barriers in a rather flat and broad energy surface, that is, support the notion that the folding free‐energy landscape of DNA‐HPs is a rugged one rather than a well‐defined two‐state system. As the concentration of α‐Syn is increased, non‐native conformations of the DNA‐HP become progressively populated. These additional conformational states are formed by strong interactions with α‐Syn, most likely through the N‐terminus of the protein and the phosphate backbone along with a weakening of base stacking by the NAC region through hydrophobic interactions (Figure 4). Furthermore, the pressure‐dependent measurements reveal strong and compact (void‐free) binding of α‐Syn to the DNA‐HP. Moreover, the fact that the population of the protein‐bound state does not increase with pressure, which opens up the stem region, suggests that the protein binds to the loop region of the DNA‐HP.

**Figure 4:**
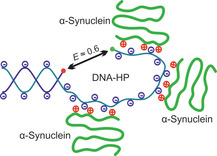
Schematic representation of the proposed interaction between the intrinsically disordered α‐Syn and the DNA‐HP, which is most likely dominated by charge‐charge interactions of the DNA and the N‐terminal domain I of α‐Syn.

In the presence of the ATPS, stabilization of the altered protein‐bound DNA‐HP conformation is observed, reaching population levels of approximately 90 % of the overall distribution (Figure 3). This might be due to the fact that the conformational subspace of the DNA‐HP becomes strongly restricted in the phase‐separated droplet phase. Owing to the strong excluded volume effect and possibly also additional enthalpic interactions with the constituents of the condensate, conformational fluctuations of the DNA‐HP become restricted, favoring compact states and hence a decrease of the population of the open state. Upon pressurization, the distribution of conformations remains unchanged, reflecting the notion that compact conformations with high packing efficiency and hence small partial volumes are favored.

In conclusion, smFRET data as shown here are able to provide a unique spectral signature for capturing local conformational changes, thereby enabling one to decipher non‐specific interactions between nucleic acids and proteins. Furthermore, we think that such approach using smFRET spectroscopy in concert with pressure modulation in studies of DNA–protein interactions, in the absence and presence of liquid condensates induced by LLPS, provide a valuable tool to infer a basic comprehension of hidden mechanisms of cell science. They can help to explore the conformational and free energy landscape of biomolecular systems including the existence of conformational substates induced by changing the solution conditions, that is, the cellular milieu, which are not easily accessible by other means. Notably, learning about pressure effects on such biomolecular assemblies can also help us to gain a better appreciation of pressure effects on DNA–protein interactions and gene regulation in general, as, for example, relevant for a better understanding of the physiology of organisms thriving at multi‐hundred bar pressures in the deep sea.

## Conflict of interest

The authors declare no conflict of interest.

## Supporting information

As a service to our authors and readers, this journal provides supporting information supplied by the authors. Such materials are peer reviewed and may be re‐organized for online delivery, but are not copy‐edited or typeset. Technical support issues arising from supporting information (other than missing files) should be addressed to the authors.

Supplementary
