# Supplementary material for: Alteration of the Conformational Dynamics of a DNA Hairpin by α‐Synuclein in the Presence of Aqueous Two‐Phase Systems
Source: Chemistry. 2020 Aug 6;26(48):10987–91. doi: 10.1002/chem.202002119 (PMC7496936; doi:10.1002/chem.202002119)
Supplement: Supplementary file 1 — Supplementary [file CHEM-26-10987-s001.pdf]

Chemistry–A European Journal

Supporting Information

Alteration of the Conformational Dynamics of a DNA Hairpin by α-Synuclein in the Presence of Aqueous Two-Phase Systems

Sanjib K. Mukherjee+, Jim-Marcel Knop+, Simone Möbitz, and Roland H. A. Winter*[a]

Supporting Information

Table of Contents

S.1 Experimental and Methods Section
S.2 Supporting References
S.3 Supporting Figures

S.1 Experimental and Methods Section

Materials and sample preparation

Dextran 10 kDa was purchased from Carl Roth GmbH + Co. KG (Karlsruhe, Germany). Polyethylene glycol (PEG) 4.6 kDa was obtained from Sigma-Aldrich (Steinheim, Germany) and was used as received without further purification. Dextran 10 kDa labeled with rhodamine B was purchased from Invitrogen (Thermo Fischer Scientific). As described by Baltierra-Jasso et al.,[1] the DNA oligonucleotides were synthesized and fluorescently labeled by IBA life solutions GmbH (Goettingen, Germany). The sequences of the two DNA strands used to prepare the DNA hairpin are: H2 strand: 5'-TGG CGA CGG CAG CGA GGC TTA GCG GCA AAA AAAAAAAAAAAAAAAAAAAAAAAAAAAA AGC CGC X-3' (here, X is T-Atto 550); A2 strand: 5'-GCC TCG CYG CCG TCG CCA-3' (here, Y is T-Atto 647N). For annealing, both, H2 and A2 strands were first mixed in a 1:1 molar ratio in buffer solution containing 20 mM TrisHCl, 50 mM NaCl (pH = 8.0) and annealed by first heating at 95 °C for 5 min, followed by gradually cooling down to room temperature at a rate of -0.5 °C/min using a thermocycler. The buffer solution used in the measurements contains 20 mM TRIS (pH 7.4) and 15 mM NaCl and was filtered by a 0.45 µm sterile Whatman Puradisc 30 syringe filter. Square-shaped flexible fused silica capillaries (WWP 050375, Polymicro Technologies) were procured from CM Scientific (UK). The 2-component epoxy glue (UHU plus endfest 300) was obtained from UHU GmbH & Co. KG. (Germany). The pressure plug is made of a piece of high pressure tube TBG-100-4 provided by Nova Swiss (Switzerland).

Characterization of the ATPS

The ATPS system used in our study consists of PEG 4.6 kDa (polyethylene glycol) and dextran 10 kDa, a glucose polymer, and has been characterized in detail und used for various biochemical applications before,[2-4]After determination of the binodal for PEG (4.6 kDa) and dextran (10 kDa) in our buffer used, the composition of the ATPS chosen for the smFRET measurements was set to 11 wt% PEG and 11wt% dextran.

Expression and purification of α-synuclein

The expression and purification of α-synuclein was carried out as described.[5,6] The plasmid pT7-7 expressing human α-synuclein was transformed into *Escherichia coli* strain BL21 (DE3). A single colony was picked and inoculated into 100 mL LB medium containing 150 µg/mL ampicillin and grown at 37 °C with shaking at 250 rpm until the absorbance at 600 reached 0.8. Induction was then carried out by adding 1 mM IPTG (final concentration) and the culture was further grown under similar conditions for 3 h. The cells were harvested and resuspended in 0.75 mL of buffer (50 mM Tris-HCl, pH 7.5, 10 mM EDTA and 150 mM NaCl) and frozen at -80 °C. Tubes containing frozen cells were placed in a boiling water bath for 7 min and the supernatant collected after centrifugation at maximum speed for min. Streptomycin sulfate (136 µL/mL of supernatant) and glacial acetic acid (228 µL/mL of supernatant) were added and centrifuged for 2 min. Again, the supernatant was recovered and precipitated with ammonium sulfate (saturated ammonium sulfate at 4°C was used 1:1, v/v, with supernatant). The protein was collected as a precipitate by centrifugation and washed once with 1 mL of ammonium sulfate solution (4 °C, 1:1, v/v, saturated ammonium sulfate and water). The washed pellet was resuspended in 900 µL of 100 mM ammonium acetate (to form a cloudy solution) and precipitated by adding an equal volume of ethanol at

room temperature. Precipitation with ethanol was repeated once more. The pellet was resuspended in 100 mM ammonium acetate and extensively dialyzed against 10 mM Tris-HCl buffer, pH 7.4.

Single-molecule FRET (smFRET) measurements

SmFRET measurements were carried using a confocal fluorescence microscope (MicroTime 200, PicoQuant) under freely diffusing conditions. The pulsed interleaved excitation (PIE) FRET technique was used to separate dually labeled from singly labeled species.[7] Briefly, in the PIE FRET technique, both the donor and acceptor are alternatively excited by the laser pulse. First, a laser pulse of suitable wavelength excites the donor and then another laser pulse excites the acceptor independently from FRET after a certain time delay (50 ns, 20 MHz repetition rate), allowing us to calculate the photon stoichiometry, S, which is the ratio of photons emitted after donor excitation and the sum of total photons emitted after donor and direct acceptor excitation. For a donor-only species, $S = 1$, and for an acceptor-species only, $S = 0$. A green laser pulse at 560 nm (LDH series, PicoQuant) and a red laser pulse at 635 nm (LDH series, PicoQuant) was used to excite the donor Atto 550 and acceptor Atto 647 N, respectively. A quad band dichroic mirror (ZT 405/488/561/640, Chroma) was used to reflect both the green and red laser light to the entrance port of the fluorescence microscope. Donor and acceptor fluorescence signals were separated to two different detection channels, first by using a dichroic mirror (FF 650 Di01, Semrock), followed by band pass filters FF 01-593/40 (Semrock) and FF 01-676/29 (Semrock). Two SPCM-AQR series single photon avalanche diodes (SPAD) were used as detection channels for the donor and acceptor fluorescence.

High pressure fluorescence cell for smFRET measurements

A square-shaped silica microcapillary based system was used as high-pressure cell for the smFRET measurements.[8,9] It serves both as optical window and mechanical body. By using a square-shaped microcapillary in the present study instead of a cylindrical shaped capillary, artifacts arising from the curved surface of a cylindrical capillary are avoided. The polyamide-coated capillary with inner and outer diameters of 360 and 50 µm, respectively, has about the same thickness as a standard microscope cover slip and provides a flat window surface, and can withstand pressures up to about 1500 bar. The capillary was cut to a length of 30 cm and then glued into a pressure plug (1.6 mm hole diameter) by using a two-component epoxy glue, which was connected to conventional pressure tubing (Nova Swiss) using a metal coupling unit. The polyamide coating of the capillary was burned away at the location of the optical path. After sucking the sample solution into the capillary, the free end of the capillary was flame sealed. The capillary was then fixed on an aluminium stage, whose position can be controlled with high accuracy in all three dimensions, allowing fine tuning of the focal position. A manual hand pump was used to generate the pressure. Silicone oil was used to separate the sample solution from the pressurizing medium (water).

S.2 References

1. L. E. Baltierra-Jasso, M. J. Morten, L. Laflör, S. D. Quinn, S. W. Magennis, *J. Am. Chem. Soc.* **2015**, *137*, 16020–16023.
2. M. S. Long, C. D. Jones, M. R. Helfrich, L. K. Mangeney-Slavin, C. D. Keating, *Proc. Natl. Acad. Sci. U.S.A.* **2005**, *102*, 5920-5925.
3. L. Arns, R. Winter, *Chem. Comm.* **2019**, *55*, 10673-10676.
4. R. Oliva, S. Banerjee, H. Cinar, R. Winter. *Chem. Comm.***2020**, *56*, 395-398.
5. M. J. Volles, P. T. Lansbury, *J. Mol. Biol.* **2007**, *366*, 1510-1522.
6. R. Shaltiel-Karyo, M. Frenkel-Pinter, N. Egoz-Matia, A. Frydman-Marom, D. E. Shalev, D. Segal, E. Gazit, *PLoS One* **2010**, *5*, e13863.
7. S. Ruettinger, V. Buschmann, B. Kraemer, S. Orthaus, F. Koberling, *Appl. note, PicoQuant GmbH*, 2013.
8. J. D. M¨uller, E. Gratton, *Biophys. J.* **2003**, *85*, 2711–2719.
9. S. Patra, C. Anders, N. Erwin, R. Winter, *Angew. Chemie. Int. Ed.* **2017**, *56*, 5045–5049.

S.3 Supporting Figures

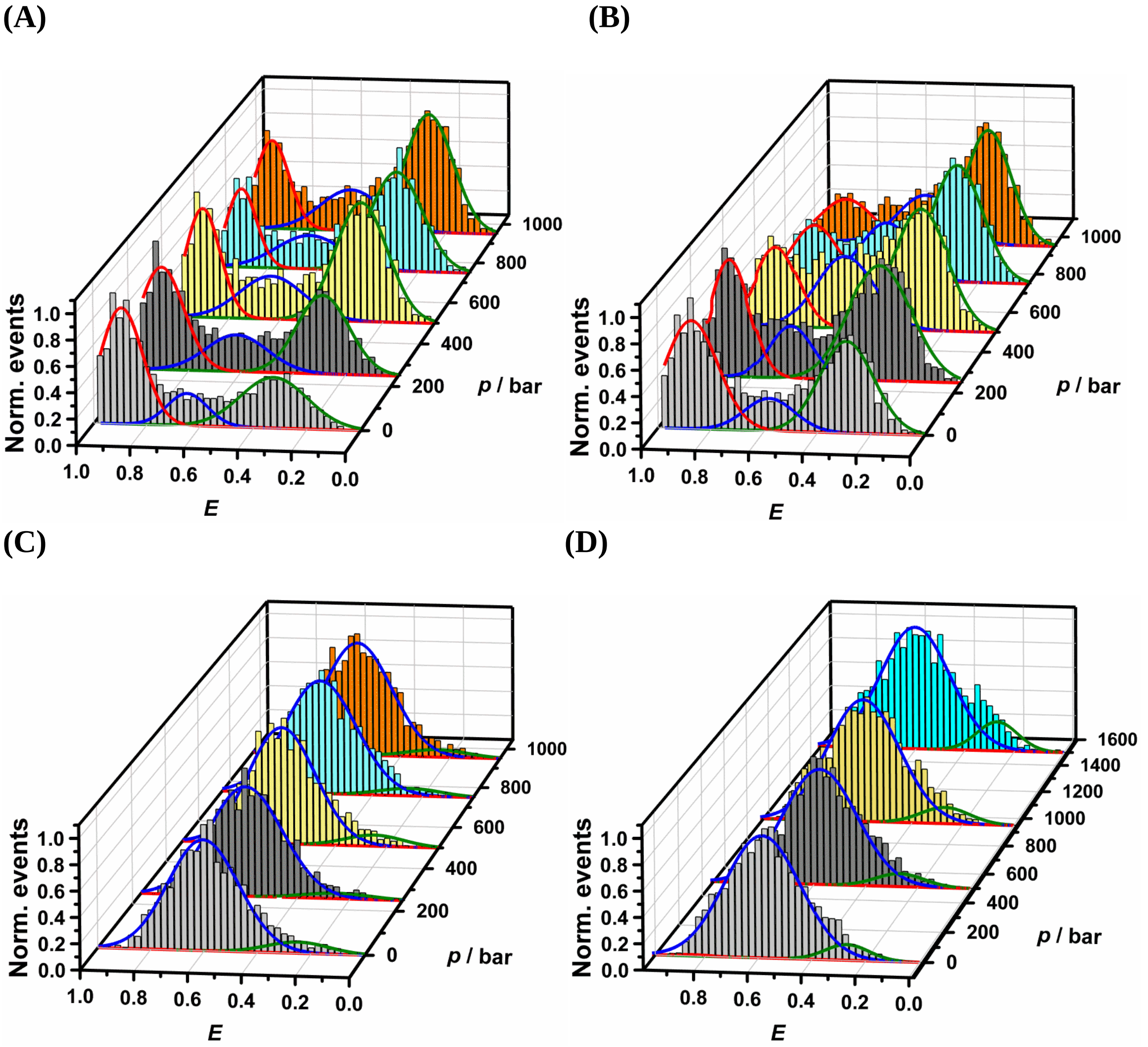

Figure S1. SmFRET histograms of the DNA-HP with Gaussian fits as a function of pressure, (A) and (C) with 50 µM α-Syn, and (B) and (D) with 150 µM α-Syn. (C) and (D) containing 11 wt% PEG and 11 wt% Dextran (ATPS). Buffer conditions in all measurements were 20 mM Tris/HCl pH 7.5, 15 mM NaCl, 25 °C.

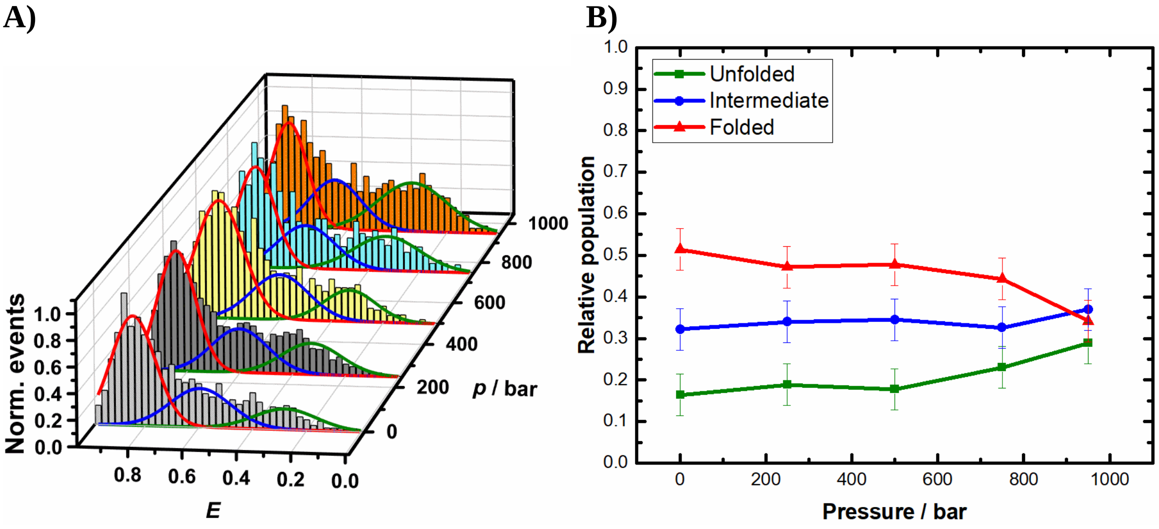

Figure S2. (A) SmFRET-histograms of the DNA-HP in 11% PEG as a function of pressure in the presence of 50 µM monomeric α-Syn. (B) Population of different conformational states of the DNA-HP in 11% PEG as a function of pressure in the presence of 50 µM monomeric α-Syn.

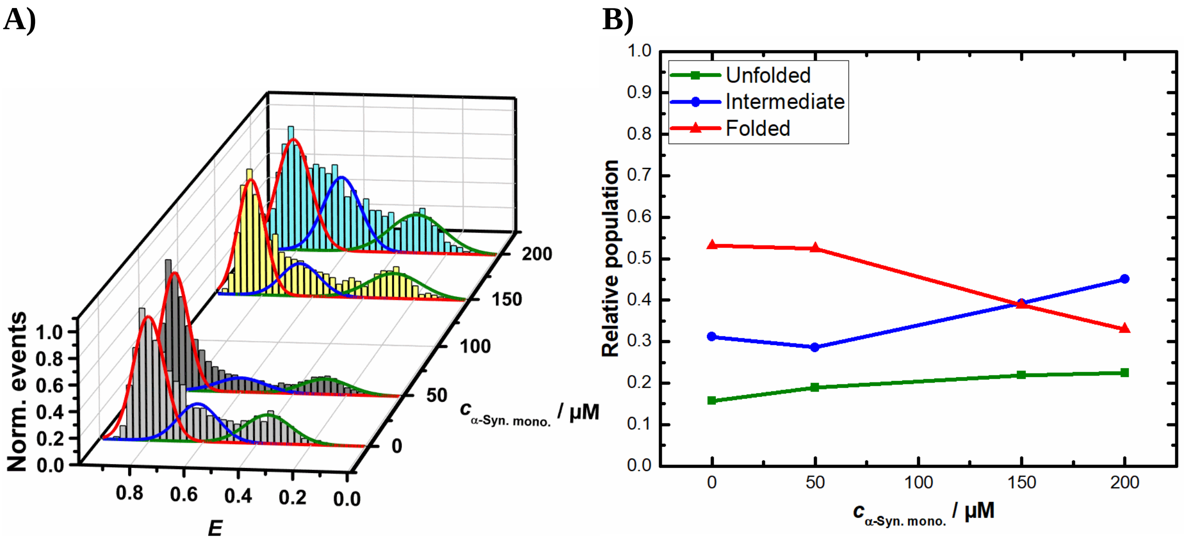

Figure S3. (A) SmFRET-histograms of the DNA-HP in 30% dextran as a function of α-Syn concentration. (B) Population of different conformational states of the DNA-HP in 30% dextran as a function of α-Syn concentration.
